# Supplementary material for: Metagenomic detection of eumycetoma causative agents from households of patients residing in two Sudanese endemic villages in White Nile State
Source: PLoS Negl Trop Dis. 2022 Aug 30;16(8):e0010385. doi: 10.1371/journal.pntd.0010385 (PMC9467367; doi:10.1371/journal.pntd.0010385)
Supplement: Attachment — Submitted filename: PNTD-D-22-00419-comments v1.0.docx [file pntd.0010385.s002.docx]

PNTD-D-22-00419

General comments: Great overall idea and performance. Requires heavy editorial action.

General questions:

It is not clear to me in multiple passages which environmental samples we are talking about (i.e. from endemic v non-endemic villages/locations). This is especially important when making the distinction between environmental samples and making the case that eumycetoma pathogens came from a specific location.

Generally, most fungal isolates from both locations are not associated with eumycetoma. This may be difficult to assay as association is culture/metagenomics-based and this evaluation provides good evidence that many eumycetoma-associated genera do not like to grow in culture.

For fungal cultures: eumycetoma is often considered an invasive fungal infection (i.e. it grows in tissue). Would be worth reconsidering culture protocol specifics (i.e. direct inoculation into agar following cutting of agar with sterile blade, use of more nutrient-poor selective PDA v SAB to promote sporulation, tissue samples v black grain samples, etc.) to optimize recovery of these molds as is financially and clinically feasible for future studies or for discussion in this submission.

Interesting that the eumycetoma pathogens were not particularly associated with thorns. Would be worth expounding on how exactly the environmental samples that contained said pathogens would be traumatically implanted in the patients considered v other means of inoculation.

Would also be worth commenting on the slight differences in metagenomics between this and the previous study (i.e. *A.alternatum* and *F.senegalensis* issue) and that *Alternaria alternatum* was actually recovered in environmental samples (at least as text is written currently, somewhat confusing).

Are tables to be published as base excel files or will these be formatted? Ideally should be formatted as, while complete, these tables are somewhat unfriendly to readers in design and breadth.

Editorial actions:

Title:

No s required after metagenomics (i.e. noun v adverb for detection).

Suggest changing “causative” to “eumycetoma-associated agents.” This reflects the full text and limitations of culture association with environmental molds better.

Suggest changing to “from households of patients residing in two Sudanese endemic villages.” Easier to read and vague enough to capture the entirety of your evaluation.

No need for comma before “in.” Not a clause.

Abstract:

First sentence:

“endemic” to not “endemic” in.

Suggest removing comma and either ending sentence or changing “and” to “with” and “has” to “featuring.” i.e. Eumycetoma is a chronic debilitating fungal disease endemic to tropical and subtropical regions with Sudan featuring the highest eumycetoma incidence.

Second sentence:

Suggest rewording for ease of read/conveyance: Ex: Among the 50 species of fungi most commonly associated with eumycetoma *Madurella mycetomatis* is often referenced as the most common pathogen.

Third sentence:

Suggest Oxford comma following “features.” Suggest reporting final segment of list. Ex: There is an enormous knowledge gap related to this neglected disease and its pathogenesis, epidemiological features, and host-specific factors that could contribute to either.

Fourth sentence:

Suggest removing “some” and replacing with “a group” or “a subset.”

Fifth-Seventh sentences:

Suggest merging. Ex: Utilizing a metagenomics approach and samples collected from clinical black grains (BG) and common household environments we aimed to assay both the habitat of eumycetoma-associated fungi and its possible connection with eumycetoma patients living in two different eumycetoma endemic villages within the White Nile State of Sudan.

Eighth-ninth sentence:

Suggest rewording/merging: Ex: DNA sequencing targeting the fungal ITS2 domain was performed on samples of soil, animal dung, housing walls and roofs, and *Acacia*-species thorn samples and compared with culture-dependent methods of fungal isolation.

Would also add addition sentence here to reference comparison with non-endemic locations. Ex: A similar protocol was performed on samples obtained from non-endemic villages/locations including….

Eleventh sentence:

Suggest changing “and that” to “which.”

To a lesser extent rather than in a lesser extent.

12^th^ sentence:

Suggest changing “and that” to “which.”

14^th^ sentence:

Suggest changing to “most common” i.e. “first most common” and “third most common.”

Suggest dropping houses and changing to “roof” i.e. In contrast, it was not detected in the roof or thorn samples nor in the soils from non-endemic regions.

15^th^ sentence:

Unclear connection being drawn here. Which environmental samples? And which fungal species? Presumably this is meant to connect BG sample molds to environmental sample molds from endemic villages. Ex: *M.mycetomatis* and other eumycetoma-associated fungal species were identified in both BG samples and endemic household samples utilizing this protocol. Two notable exceptions were found with *Acremonium alternatum* and *Falciformispora senegalensis* which were recovered in BG samples but not household samples. This may suggest that these specific infections were acquired outside of the studied endemic villages.

16-17th sentence:

Suggest connecting this to your previous findings listed in the abstract. Ex: Taken together, commonalities amongst the ASVs observed in both BG and environmental samples in the studied endemic villages suggest that eumycetoma is likely acquired through environmental exposure. Hence risk factors associated with higher exposure to these pathogens in the environment including through housing made from local soil and animal dung, poor sanitation, and the common practice of walking barefoot likely contribute to the higher incidence of eumycetoma observed in these villages.

Author summary:

First sentence (line 76):

Suggest changing “at the” to “within the.”

Second sentence (lines 77-80)

Suggest rewording for clarity and brevity. Ex: Although never isolated in culture, *M.mycetomatis* represented both the most common eumycetoma-associated fungus found within soil samples and the third most common found within dung and housing wall samples.

Ideally would couple this with third sentence (line 81). Ex: Further of interest was that *M.mycetomatis* was not isolated from roof or Acacia-species thorn samples.

Fourth sentence (line 81)

Suggest removing “the” as is not necessary.

Fifth sentence (lines 85-87)

Suggest keeping present tense (or whatever tense PLOS NTD publication prefers, unclear from guidelines).

Body:

Introduction:

First sentence (lines 97-99):

Suggest slight reword for clarity and read. Ex: depending on whether the causative agent is fungal or actinobacteria respectively.

Second sentence (lines 99-101):

Suggest rewording for read and grammar. Ex: Eumycetoma is more common between the two diseases. In Sudan it accounts for an estimated 70% of all mycetoma cases.

Third sentence (lines 101-103):

Suggest rewording for read and grammar. Ex: Mycetoma is generally endemic to tropical and subtropical climates particularly in areas featuring moderate aridity, low humidity, and a short rainy season.

Fourth sentence (lines 103-106):

Repeated “the” is not necessary in listing. Ex: A recent study found that aridity, proximity to water sources, lower soil concentrations of sodium and calcium, and the distribution of thorn-bearing trees served as the strongest predictors of mycetoma in Sudan.

Fifth sentence (line 106):

Would be clear about “the models.” Ex: The statistical models utilized in this study predicted the highest rate of occurrence….

Lines 114-115:

*Sordariales* (a fungal order) is generally italicized.

Would separate into two sentences. Ex: Worldwide the main causative agent is *Madurella mycetomatis* from the order *Sordariales*. This organism is commonly associated with the production of black grains within affected tissues.

Line 118:

Suggest reword for read and grammar. Ex: Other fungi associated with black grain eumycetoma are commonly from the order *Pleosporales* including…

Lines 118-122:

Commas after each order unnecessary. Suggest replacing with “which.” Ex: In contrast, pale grain eumycetoma species are typically from the order *Hypocreales* including *Acremonium* spp, *Cylindrocarpon* spp, and *Fusarium* spp or from the orders *Eurotiales* or *Microascales* including *Aspergillus* spp and *Microascus gracilis* respectively.

Lines 124-127:

Suggest rewording for clarity. Split into two sentences ideally. Ex: Eumycetoma generally responds poorly to the few currently available antifungal therapies all of which can be toxic, expensive, or generally unavailable within many mycetoma endemic regions. It may then be unsurprising that aggressive surgery up to and including amputation is often the only recourse for treating this disease in those areas.

Lines 128-130:

Suggest slight reword for clarity of second sentence. Ex: This difficulty can likely at least in part be attributed to lack of clarity regarding mycetoma pathogenesis, epidemiology, and the specific host factors that might contribute to either.

Line 132:

Aetiologic and causative are mutually redundant. Would pick whichever author prefers.

Line 133:

Thorn should be plural (thorns).

*Acacia* should be italicized as a genus name.

Line 134:

“The” before “mammal dung” is unnecessary.

Line 136:

*Sordariales*, as a fungal order, should be italicized.

Line 137-138:

“Still” is unnecessary in this sentence.

Lines 140-143:

Suggest rewording for clarity. Ex: A recent study utilizing ITS2 metagenomics demonstrated the presence of multiple fungal species most commonly associated with soil and animal dung, including eumycetoma-associated agents, within BG samples obtained from patients living within the eumycetoma-endemic region of the White Nile.

Lines 147-151:

Suggest rewording for clarity. Ex: To clarify the specifics of the epidemiology and environmental niche of the fungal “consortia” found within BG samples we undertook a study of soil and household samples from patients diagnosed and confirmed to have eumycetoma utilizing a similar metagenomic approach.

Materials and Methods:

Lines 162-164:

Suggest adding reference to previous method utilized to identify eumycetoma pathogen (per Table 1 was metagenomics or culture but should be in body for clarity).

Line 169-173:

Suggest rewording for clarity and grammar. Important to maintain plurality with samples when listing and to italicize *Acacia*. Ex: Environmental samples were obtained from superficial soil (N=9) and soil at 30 cm depth (N=9), housing samples from walls (mostly made of soil and animal dung, N=7) and roofs (mostly made from local tree/plants, N=6), animal dung from domestic cows, goats, and donkeys (N=8), dried local plants generally fed to live stock (N=3), and thorns from *Acacia* species trees local to the area (N=7) (Table 1).

Lines 175-177:

Suggest rewording for grammar and clarity. Ex: Additionally, soil samples were collected from non-endemic locations including Wagga village in Kassala State (N=3) and Port Sudan within Port Sudan State (N=2) (Table 1, Fig. 1).

Lines 178-180:

Suggest rewording for grammar (many commas here but not many clauses or lists). Ex: The samples were collected utilizing sterile gloves placed into sterile labeled 50 ml Falcon tubes with a sterile spatula. Samples were then brought to the laboratory to be stored at 4 C before being portioned for both culture and DNA extraction.

Line 183:

Comma after “media” is unnecessary. Can shorten “which included” to “including.”

Line 188:

Dung and thorn can be singular.

Line 192:

Oxford comma after “mycelia.”

Line 196:

Suggest slight reword for grammar. Unclear of how “Still,” affects meaning and reading. Ex: Additional fungi unable to be identified by culture or morphology were identified by Sanger sequencing.

DNAs can be replaced with “Nucleic Acids” for clarity and grammar.

Line 199:

DNAs can be replaced with “nucleic acids” for clarity and grammar.

Line 204:

As written should replace “was” with “were” as we are speaking of multiple species.

Could also be rewritten as “definitive speciation was determined by BLAST database query.”

Line 207:

Suggest rewording “samples biodiversity” with “biodiversity within samples” to avoid confusing possessive tenses.

Line 209:

Can remove comma after “cabinet.” Would reword end of sentence for clarity. Ex: “…and then resuspended utilizing sterile water…”

Line 212-214:

This sentence is a bit confusing.

So, contamination was assayed by utilizing a blank control of DNA free water? That is, it was added as a separate sample then extracted and sequenced rather than added to a test sample (as this wording implies)?

If this is the case, would suggest slight reword. Ex: Contamination between samples was excluded using Ultrapure DNA free water as a separate blank control.

Line 222:

Can remove comma after “Library” and replace with “with.” Ex: Libraries were prepared using Nextera XT DNA Library with sequencing performed via the Illumina MiSeq Paired-End platform.

Line 228:

Oxford comma after “merged”

Line 230:

Suggest removing comma after parenthetical and adding “and”

Line 232:

Spelling is “visualization”

Line 239:

Can remove comma after “procedure”

Results:

Lines 246-248:

Can remove comma after “samples” and replaced “and they” with “which”

Oxford comma between parenthetical and “and other species”

Line 248:

Genus species shouldn’t be capitalized. Ex: *M.mycetomatis*

Lines 250-251

Can change “and that” to “which”

Oxford comma between “*Bipolaris* (1.6%)” and “and *Chaetomium atrobrunneum*”

Lines 253-256:

Suggest rewording for clarity and grammar. Ex: Several species of eumycetoma-associated fungi were found by ITS2 Sanger sequencing but not observed to grow in culture. These include…

Line 260:

Should not capitalize metagenomics

Line 263:

While controversial, all levels of taxonomy are starting to be italicized. Ex: subkingdom *Alveolata*. Should conform to NTD standards on the matter.

Line 265-267:

Suggest splitting list into two separate sentences. Ex: As shown by the alpha rarefaction analysis (Fig. 2a), samples with the highest ASV diversity were from deep soil, housing walls, and superficial soil. This was followed by animal dung, housing roofs, thorns, and finally clinical BG samples.

ASVs and BGs here should be singular for grammar.

Line 268:

ASV here should be singular.

Lines 269-270:

ASV here should be singular.

Line 273:

Spelling is visualized.

There is a missing closed parenthetical for (based on the… Would suggest adding closed parenthetical after “ASV” as that seems to be the intent of the information included in the parenthetical.

Lines 276-279:

Suggest rewording for clarity and grammar. Ex: Compared to total ASV count, ASVs of eumycetoma-associated fungi were found more commonly in housing roof (15%) and wall (13%) samples. This was followed by animal dung samples (11.6%), superficial soil and thorn samples (11.5% each), and deep soil samples (11%) (Tab. S1c).

I believe that both superficial soil and thorn samples are meant to have 11.5% each rather than 11.5% collectively but is worth clarifying in text as baseline excel tables are very unfriendly to reader.

Lines 281-285:

Suggest rewording for clarity and grammar, and to replace AVS with ASV.

Ex: ASV metagenomics of samples obtained from the environment of endemic villages identified multiple genera associated with eumycetoma including….

It is not clear per text if the environmental samples were from endemic villages, non-endemic locations, or both. Should clarify.

“in a lesser extent” should be replaced by “to a lesser extent”

Commas should separate this list after “to a lesser extent”.

It is not clear if both *Bipolaris* and *Acremonium* represented 0.01% of the ASVs associated with eumycetoma together or if both are 0.01%. Should clarify.

Lines 287-289:

I am deeply confused by this sentence.

Does overall imply that it is across both environmental and clinical samples? And across both endemic and non-endemic environmental samples? Should clarify.

I believe what is meant is something like: *M.mycetomatis* represented 10% of all eumycetoma-associated sequences (0.7% of total sequences) across all samples within the study. This places it at the third most abundant eumycetoma-associated species following *Exserohilum rostratum* (28%) and *Alternaria alternatum* (14%).

Lines 289-291:

Suggest rewording for grammar and clarity:

Ex: Interestingly of these three only *A.alternatum* were found to grow in culture (2%).

Lines 292-296:

Another confusing set of sentences.

I believe what is meant is something like: On average patients affected by eumycetoma associated with *M.mycetomatis* had more *M.mycetomatis* found within their associated environmental samples when compared with patients with eumycetoma associated with other fungal genera (4% v 1%). However, this observed difference was not statistically significant (p>0.05).

Should clarify. And add specific calculated p value ideally.

Fungi richness and diversity in soil

Line 299:

Suggest rewording for grammar. Ex: Richness of fungal diversity in soil

Line 300:

Soil should be singular

Lines 303-306:

Suggest rewording for grammar and clarity. Ex: 234 ASVs were found in superficial soil samples while 225 were found in deep soil samples with 27 (11.5%) and 25 (11.1%) being from eumycetoma-associated fungi respectively (Table S1b).

Lines 307-312:

Suggest rewording for grammar and clarity. Are the hyphens representing a range or that one side is from superficial and another deep? If so, Ex: *M.mycetomatis* was the most common eumycetoma-associated species demonstrated in both superficial (0.79%) and deep (0.64%) soil samples. For superficial and deep soil samples this was followed by *Curvularia lunata* (0.61%, 0.5%), *E.rostratum* (0.34%, 0.3%), *Cladosporium* spp. (0.31%, 0.13%), and *A.alternatum* (0.3% each) respectively (Table S1c).

Example changed to add clarity to *C.lunata* naming, correct spelling of *Cladosporium*.

Line 312:

Suggest adding “each” to parentheticals to clarify if observed percentages are for both deep and superficial. If they are a combination of both would add “together.”

Line 313:

Would suggest write out full name of *Madurella fahalii* for clarity as is first time referenced in text.

Lines 316-317:

Super interesting finding. Would consider adding a reason or speculation in the discussion about this. You would think metagenomics would have better resolution than Sanger and should be more sensitive than either Sanger or culture.

Line 321:

Soil should be singular.

Line 322:

Suggest separating sentences. Ex: …was detected in Kassala soil. In contrast to the endemic samples, ….

Line 324:

Other should be singular.

Line 326:

Spelling is *Cladosporium*

Lines 329-331:

Suggest slight rewording for grammar and clarity. And utilizing PCoA to keep abbreviations standard from start of text. Ex: Greater beta diversity was noted on PCoA analysis amongst ASVs observed in soil samples from the endemic villages in White Nile and those in Port Sudan when compared with the non-endemic village of Wagga (Fig. 3).

Fungal richness and diversity in walls and dung samples.

Line 333:

Suggest rewording for grammar and clarity. Ex: Richness of fungal diversity in wall and animal dung samples.

Line 334:

“dungs” and “walls” should both be singular.

Line 335:

“Dungs” should be singular.

Would suggest moving “of which” to after “23” to keep parallel with last clause in sentence. Ex: ….23 of which….29 of which…

Line 338:

“dungs” should be singular.

Line 341:

Spelling is *Cladosporium*

Line 342:

Suggest changing “houses walls” to “housing wall” to avoid confusing possession v plural and assist read.

Fungi richness and diversity in houses roofs and thorns samples

Line 348:

Suggest rewording for grammar and clarity. Ex: Richness of fungal diversity in housing roof and thorn samples.

Lines 349-352:

Suggest rewording for clarity and grammar. Ex:

Nine roof and seven thorn samples were studied by metagenomics. 117 ASVs were noted in roof samples with 18 (15%) of them being associated with eumycetoma while 69 ASVs were found in thorns with eight (11.6%) of them being associated with eumycetoma (Table S1b).

Suggest adding percentages to keep with general format of text. Rounding in keeping with NTD policy and author’s prior rounding conventions.

Lines 353-356:

Somewhat confusing set of sentences. I believe this is to show which genus-level and species-level IDs were found amongst fungal species ASVs in roof samples. Wording is not clear. If this is so:

“roofs” should be singular.

“species” should be changed to “genera” or “ASVs” to keep with idea of both sentences.

Would suggest changing “Among species” to “Amongst ASVs with species-level identification” for clarity.

Lines 356-358:

Similar confusion here. If idea is to demonstrate genus and species level IDs:

Suggest separate sentences. Ex: In thorn samples the only eumycetoma-associated fungus to be identified to species level was *E.rostratum* (3%). However, multiple genus-level ASVs were found including…

Lines 359-362:

Suggest rewording for grammar and clarity. Ex: Notably culture was only able to identify *A.niger*, *A.nidulans*, and *F.oxysporum* from thorn samples while *A.nidulans*, *A.niger*, *A.terreus*, and *C.atrobrunneum* were identified from roof samples. Interestingly, *C.atrobrunneum* was not observed in metagenomics analysis.

This is another super interesting finding with not finding *C.atrobrunneum* on metagenomics (presumably per text wording). Would attempt to expound on that in discussion if possible with *F.falciforme* observation above.

Lines 363-365:

This segment seems to leave a bit unsaid regarding how frequently non-eumycetoma associated ASVs were observed statistically given percentages in the texts and ASVs. Would suggest either adding passages in your other samples expounding on this point or removing this passage. Favor the former but information may not be available. Could weaken your case or could provide evidence of new associations for other genera.

Fungi richness and diversity in black grains samples.

Line 367:

Suggest rewording for grammar and clarity. Ex: Richness of fungal diversity in black grain samples.

Lines 368-373:

Suggest rewording for grammar and clarity. Ex: ITS2 amplicons previously obtained from BG samples from eumycetoma patients living in the endemic villages of Alsobahi (BG…., houses….) and El Andalous (BG…., houses….) were analyzed using the newest version of Qiime2 package….

Lines 374-376:

Suggest rewording for grammar and clarity. Ex: The new analysis demonstrated similar results to prior analysis with an older Qiime2 package and found both eumycetoma-associated agents and non-associated agents….

Lines 376-379:

Deeply confused about this passage. The second analysis found *Fusarium* not *Acremonium*? Are they not both associated with eumycetoma per the text? Worth expounding on in discussion as could be seen as a weakness to metagenomics approach. Likely inherent to two closely related fungi in terms of phylogenetics.

Suggest rewording for grammar and clarity. Ex: Interestingly, the identity of the fungi associated with one patient’s BG sample (house…) was found to be different on this reanalysis. On first pass the fungus was identified as *A.alternatum* while on second pass it was called *Fusarium* spp.

Would then address in discussion how both being in *Hypocreales* and being somewhat closely related might explain how this happened. Likely will undermine argument less than approaching it from a diversity of fungi in the sample perspective.

Lines 380-387:

Suggest rewording for grammar and clarity. Ex: All fungal species associated with eumycetoma were similarly detected on the new analysis with two notable exceptions. BG18 from a patient in house X failed to detect a previously characterized *F.senegalensis* while BG5 from a farmer in house Y failed to demonstrate a previously characterized *A.alternatum*. Further, three non-eumycetoma associated fungi were recovered in BG samples that were not found in environmental samples including *Sistotrema adnatum* (BG18), *Inocybe vulpinella*, and *Vishniacozyma* spp. (both BG5) (Table S1f, Fig. 4). Figure 4 visualizes the full range of fungi detected in BG samples.

Discussion:

Line 392:

Oxford comma after “communities”

Line 396:

Suggest either replacing “that” with “it” or adding “gap” after “that” for grammar and read.

Line 401:

Suggest separating “in general” with commas as a separate clause to parallel the second clause “,in particular,” Or removing commas from second clause.

Line 405:

This may be an issue with the copy I received but is “metagenomics” in a slightly different color for everyone else? Is this an artifact of bolding? Very minute issue but is strange and noticeable on read.

Lines 409-412:

Should be clear when speaking of environmental samples if we are talking about endemic or non-endemic context. This can affect your conclusion and could affect reader’s understanding.

Comma between “*Microascus”* and “*Bipolaris”* and Oxford comma after “*Bipolaris”*

Lines 412-414:

Suggest rewording for grammar and clarity. Ex: Amongst environmental samples from endemic villages *M.mycetomatis* represented the first most common species recovered from all soil samples and third most common from dung and housing wall samples by metagenomic analysis.

Lines 414-415:

Spelling is “findings”

Would expound here on how that data supports it and speak about the thorn negatives. That is still a sticking point to me.

Would keep eumycetoma language for clarity. This study has nothing to do with actinomycetoma.

Lines 417-420:

This is a very confusing passage. I believe you meant “The previous study demonstrated” not “The present study demonstrated”

Would reword, remove commas after “Sudan” and “river” and add “and” after “river.” Ex: The previous study demonstrated the occurrence of *M.mycetomatis* and other species associated with eumycetoma in the environment of the Southeastern villages of Sudan along the Nile River and confirmed their spatial distribution therein through regression and machine learning techniques.

Lines 423-425:

This is a very confusing passage. Multiple species of eumycetoma-associated fungi were found in environmental samples. *A.alternatum* was actually detected in multiple environmental samples from both endemic and non-endemic locales. It was not detected on the second round of metagenomics in one patient previously diagnosed with it through the first metagenomics round. Is that not accurate? While your implication is likely accurate for *F.senegalensis*, I do not believe the passage accurately represents your findings nor can it expand to *A.alternatum*.

Would keep eumycetoma language for clarity. This study has nothing to do with actinomycetoma.

Line 427:

Would suggest removing “the” before “different” for grammar.

Line 432:

ASVs not AVS

Line 433-436:

Suggest rewording for grammar and clarity. Further interestingly *Fusarium* (0.4% of ASV sequences demonstrated in environmental samples from endemic/non-endemic villages) and *Aspergillus* species (X% of ASV sequences demonstrated in environmental samples of endemic/non-endemic villages) represented the most common species recovered utilizing culture techniques. The exact explanation for this finding is not clear.

Line 438:

Would then link this paragraph with the previous one as this passage seems to try to expound on why *M.mycetomatis* was not observed in culture. Ex: One possible explanation for this observation…

Lines 443-447:

Would reword for grammar and clarity. Ex: Another possible explanation for the difficulty of *M.mycetomatis* cultivation in vitro could be its ability to produce melanin and black grains. While this ability to escape immune responses may aid in its ability in producing such chronic infections in vivo, in vitro this may produce an effect that inhibits growth and sporulation similar to a bacterial biofilm or endospore. This idea could further be supported by the observation of hyaline molds and non-melanin containing fungi being more frequently isolated from both immunocompromised and malnourished patients than their dematiaceous counterparts.

Lines 449-453:

Would reword for clarity and grammar. Ex: While the conclusions in this study may be limited by its small sample size it does offer some suggestions to reducing the disease burden felt by those living in endemic and often impoverished areas. These include using non-endemic soil and avoiding animal dung in housing construction and the use of proper footwear and gloves in agricultural and other activities that may facilitate traumatic inoculation.

Lines 454-457:

Confusing wording here. Would stick with metagenomics even if NGS was involved for clarity.

Suggest rewording last two sentences together for clarity and grammar. Ex: While metagenomics proved very useful in assaying environmental and clinical samples towards the goal of greater understanding of the connection between environmental fungi and eumycetoma pathogens implementation of such techniques are still currently limited by prohibitive financial cost.
