# Supplementary material for: Metagenomic detection of eumycetoma causative agents from households of patients residing in two Sudanese endemic villages in White Nile State
Source: PLoS Negl Trop Dis. 2022 Aug 30;16(8):e0010385. doi: 10.1371/journal.pntd.0010385 (PMC9467367; doi:10.1371/journal.pntd.0010385)
Supplement: Attachment — Submitted filename: PNTD-D-22-00419-comments answers.docx [file pntd.0010385.s003.docx]

**Dear editor,**

Thank you very much for letting our manuscript " Metagenomics detection of eumycetoma causative agents within eumycetoma patient’s household environment in two Sudanese endemic villages, in White Nile State." (PNTD-D-22-00419) ‘’ being reviewed by the Journal of PLOS neglected tropical disease . In this rebuttal, we will answer and address the concerns raised by the reviewers on our manuscript.

We are so grateful for the reviewer’s time and effort in providing these valuable edits and comments which were of great help to us in improving our manuscript.

With kind regards,

Prof. Ahmed Hassan Fahal on behalf of all co-authors

Major Changes

The revised manuscript has now a new title: “Metagenomic detection of eumycetoma causative agents from households of patients residing in two Sudanese endemic village in White Nile State” as suggested by the second reviewer. We better explain the origin of the environmental samples under study and added additional analysis between endemic and not endemic soil samples with statistical analysis added in Table S1g), The presence of eumycetoma associated species in the soil samples was evaluated by alpha and beta diversity as shown in new Fig 2 and Fig3 including Alfa diversity with rarefaction curves of the soil samples (Fig 2b)

PNTD-D-22-00419

**Reviewer #1:**

The manuscript metagenomics detection of eumycetoma causative agents is an interesting study that adds to the existing literature concerning possible environmental niches and routes of acquisition of this devastating neglected tropical disease. Although the study size (sample numbers) is relatively modest, this is comprehensible given the laborious nature of the approaches used. The methods employed are appropriate for the study aim, and the experiments appear to have been well-conducted. On the whole, the data support the conclusions drawn by the authors, and are presented in a clear fashion. My only significant criticism is of the English usage/grammar. The entire manuscript would benefit form an extensive revision by a native English speaker.

Comment: the manuscript was carefully revised for English grammar also by a native speaker

**Reviewer 2**

Reviewer #2: Yes to all the above except as noted in attachment

General comments: Great overall idea and performance. Requires heavy editorial action.

General questions:

It is not clear to me in multiple passages which environmental samples we are talking about (i.e. from endemic v non-endemic villages/locations). This is especially important when making the distinction between environmental samples and making the case that eumycetoma pathogens came from a specific location.

Comment: Environmental samples were from 9 houses of 2 villages of the While Nile region, endemic for eumycetoma ( El Andalous n=4 and Alsobahi n=5). In 8 of these houses 8 cases of Eumycetoma were previously described, 5 of which by ITS2 metagenomics (18). Just 5 soil samples from not endemic villages from Port Sudan (n=2) and Kassala (n=3) regions were analyzed and compared to soil samples of endemic villages. In this revised manuscript we added alpha and beta diversity comparing samples from endemic and not endemic soil. As requested, we apply statistical analysis (ANOVA One-Way Analysis of Variance plus Tukey's HSD) comparing each eumycetoma causative species found in endemic and non-endemic soil.

Generally, most fungal isolates from both locations are not associated with eumycetoma. This may be difficult to assay as association is culture/metagenomics-based and this evaluation provides good evidence that many eumycetoma-associated genera do not like to grow in culture.

For fungal cultures: eumycetoma is often considered an invasive fungal infection (i.e. it grows in tissue). Would be worth reconsidering culture protocol specifics (i.e. direct inoculation into agar following cutting of agar with sterile blade, use of more nutrient-poor selective PDA v SAB to promote sporulation, tissue samples v black grain samples, etc.) to optimize recovery of these molds as is financially and clinically feasible for future studies or for discussion in this submission.

Comment: As you suggested we already tried to culture the BGs (18) and here environmental samples, using different media (see MM). Many eumycetoma-associated species including *Madurella mycetomatis* do not like to grow in culture, so that other alternative methods of eumycetoma diagnosis are being tested. We recently described by metagenomics that a consortium of several environmental fungi species were identified from the black grains samples of eumycetoma patients, of which only one species was highly predominant over the others and corresponded to the species isolated from black grain culture, when growth was possible

Mycetoma Research center (MRC) has a good fungi culture experience, we think that the growth of the eumycetoma causative specie could be inhibited by those “fungi population” introduced by trauma from the environment.

Interesting that the eumycetoma pathogens were not particularly associated with thorns. Would be worth expounding on how exactly the environmental samples that contained said pathogens would be traumatically implanted in the patients considered v other means of inoculation.

Comment: The soil, in which the dry feces of animals living in close contact with people abound, forms the floor of Sudanese rural houses. It is easy to think that these fungi-rich materials (soil, animal dunks and walls) can be inoculated with trauma during daily work, children's play or other activity as agriculture. Results suggested that the thorns are most probably fungi vectors and not the source of eumycetoma species.

Would also be worth commenting on the slight differences in metagenomics between this and the previous study (i.e. *A.alternatum* and *F.senegalensis* issue) and that *Alternaria alternatum* was actually recovered in environmental samples (at least as text is written currently, somewhat confusing).

Comment: Sorry there was a mistake, we extend the name of species *Acrenomium alternatum* and *Alternata alternata* in full, to avoid confusion.

On first pass the fungus responsible of eumycetoma in patient 5 was identified as *Fusarium spp* [18], while on second pass it was called *Acremonium alternatum* (submitted on database in date 26-may-2021_[PRJNA732838](https://www.ncbi.nlm.nih.gov/bioproject/732838)), *Fusarium spp* and *Acremonium spp* belong to the Hypocreales order, so probable none of *Acremonium spp* sequences were present in the Metagenomics database during the first analysis.

These 2 eumycetoma associated species were detected in BG and not in the environmental samples of endemic region, as well as in not endemic, just few sequences of *Acremonium persicum* were detected,

While *Alternaria alternata* was detected in both endemic and not endemic villages

Are tables to be published as base excel files or will these be formatted? Ideally should be formatted as, while complete, these tables are somewhat unfriendly to readers in design and breadth.

The Table is a Supplementary Table, consultable just online for the big quantity of data.

We tried to simplify it, adding in the legends dismissed acronym’ names. We also added an additional xls sheet (Table S1g) with statical analysis

Editorial actions:

Title:

No s required after metagenomics (i.e. noun v adverb for detection).

Comment: Done

Suggest changing “causative” to “eumycetoma-associated agents.” This reflects the full text and limitations of culture association with environmental molds better.

Suggest changing to “from households of patients residing in two Sudanese endemic villages.” Easier to read and vague enough to capture the entirety of your evaluation. Done

No need for comma before “in.” Not a clause.

Abstract:

First sentence:

“endemic” to not “endemic” in.

Comment: Done

Suggest removing comma and either ending sentence or changing “and” to “with” and “has” to “featuring.” i.e. Eumycetoma is a chronic debilitating fungal disease endemic to tropical and subtropical regions with Sudan featuring the highest eumycetoma incidence.

Comment: Done

Second sentence:

Suggest rewording for ease of read/conveyance: Ex: Among the 50 species of fungi most commonly associated with eumycetoma *Madurella mycetomatis* is often referenced as the most common pathogen.

Comment: Done

Third sentence:

Suggest Oxford comma following “features.” Suggest reporting final segment of list. Ex: There is an enormous knowledge gap related to this neglected disease and its pathogenesis, epidemiological features, and host-specific factors that could contribute to either.

Comment: Done

Fourth sentence:

Suggest removing “some” and replacing with “a group” or “a subset.”

Comment: Done

Fifth-Seventh sentences:

Suggest merging. Ex: Utilizing a metagenomics approach and samples collected from clinical black grains (BG) and common household environments we aimed to assay both the habitat of eumycetoma-associated fungi and its possible connection with eumycetoma patients living in two different eumycetoma endemic villages within the White Nile State of Sudan.

Comment: Done

Eighth-ninth sentence:

Suggest rewording/merging: Ex: DNA sequencing targeting the fungal ITS2 domain was performed on samples of soil, animal dung, housing walls and roofs, and *Acacia*-species thorn samples and compared with culture-dependent methods of fungal isolation.

Comment: Done

Would also add addition sentence here to reference comparison with non-endemic locations. Ex: A similar protocol was performed on samples obtained from non-endemic villages/locations including….

Comment: Done

Eleventh sentence:

Suggest changing “and that” to “which.”

Comment: Done

To a lesser extent rather than in a lesser extent.

Comment: Done

12^th^ sentence:

Suggest changing “and that” to “which.”

Comment: Done

14^th^ sentence:

Suggest changing to “most common” i.e. “first most common” and “third most common.”

Comment: Done

Suggest dropping houses and changing to “roof” i.e. In contrast, it was not detected in the roof or thorn samples nor in the soils from non-endemic regions.

Comment: Done

15^th^ sentence:

Unclear connection being drawn here. Which environmental samples? And which fungal species? Presumably this is meant to connect BG sample molds to environmental sample molds from endemic villages. Ex: *M.mycetomatis* and other eumycetoma-associated fungal species were identified in both BG samples and endemic household samples utilizing this protocol. Two notable exceptions were found with *Acremonium alternatum* and *Falciformispora senegalensis* which were recovered in BG samples but not household samples. This may suggest that these specific infections were acquired outside of the studied endemic villages.

Comment: Yes, this meant to connect BG sample molds to environmental sample molds from endemic villages. We changed following your suggestions

16-17th sentence:

Suggest connecting this to your previous findings listed in the abstract. Ex: Taken together, commonalities amongst the ASVs observed in both BG and environmental samples in the studied endemic villages suggest that eumycetoma is likely acquired through environmental exposure. Hence risk factors associated with higher exposure to these pathogens in the environment including through housing made from local soil and animal dung, poor sanitation, and the common practice of walking barefoot likely contribute to the higher incidence of eumycetoma observed in these villages.

Comment:

Author summary:

First sentence (line 76):

Suggest changing “at the” to “within the.”

Comment: Done

Second sentence (lines 77-80)

Suggest rewording for clarity and brevity. Ex: Although never isolated in culture, *M.mycetomatis* represented both the most common eumycetoma-associated fungus found within soil samples and the third most common found within dung and housing wall samples.

Comment: Done

Ideally would couple this with third sentence (line 81). Ex: Further of interest was that *M.mycetomatis* was not isolated from roof or Acacia-species thorn samples.

Fourth sentence (line 81)

Suggest removing “the” as is not necessary.

Comment: Done

Fifth sentence (lines 85-87)

Suggest keeping present tense (or whatever tense PLOS NTD publication prefers, unclear from guidelines).

Comment: OK

Body:

Introduction:

First sentence (lines 97-99):

Suggest slight reword for clarity and read. Ex: depending on whether the causative agent is fungal or actinobacteria respectively.

Comment: Done

Second sentence (lines 99-101):

Suggest rewording for read and grammar. Ex: Eumycetoma is more common between the two diseases. In Sudan it accounts for an estimated 70% of all mycetoma cases.

Comment: Done

Third sentence (lines 101-103):

Suggest rewording for read and grammar. Ex: Mycetoma is generally endemic to tropical and subtropical climates particularly in areas featuring moderate aridity, low humidity, and a short rainy season.

Comment: Done

Fourth sentence (lines 103-106):

Repeated “the” is not necessary in listing. Ex: A recent study found that aridity, proximity to water sources, lower soil concentrations of sodium and calcium, and the distribution of thorn-bearing trees served as the strongest predictors of mycetoma in Sudan.

Comment: Done

Fifth sentence (line 106):

Would be clear about “the models.” Ex: The statistical models utilized in this study predicted the highest rate of occurrence….Comment: Done

Lines 114-115:

*Sordariales* (a fungal order) is generally italicized.

Comment: Done

Would separate into two sentences. Ex: Worldwide the main causative agent is *Madurella mycetomatis* from the order *Sordariales*. This organism is commonly associated with the production of black grains within affected tissues.

Comment: Done

Line 118:

Suggest reword for read and grammar. Ex: Other fungi associated with black grain eumycetoma are commonly from the order *Pleosporales* including…

Comment: Done

Lines 118-122:

Commas after each order unnecessary. Suggest replacing with “which.” Ex: In contrast, pale grain eumycetoma species are typically from the order *Hypocreales* including *Acremonium* spp, *Cylindrocarpon* spp, and *Fusarium* spp or from the orders *Eurotiales* or *Microascales* including *Aspergillus* spp and *Microascus gracilis* respectively.

Comment: Done

Lines 124-127:

Suggest rewording for clarity. Split into two sentences ideally. Ex: Eumycetoma generally responds poorly to the few currently available antifungal therapies all of which can be toxic, expensive, or generally unavailable within many mycetoma endemic regions. It may then be unsurprising that aggressive surgery up to and including amputation is often the only recourse for treating this disease in those areas.

Comment: Done

Lines 128-130:

Suggest slight reword for clarity of second sentence. Ex: This difficulty can likely at least in part be attributed to lack of clarity regarding mycetoma pathogenesis, epidemiology, and the specific host factors that might contribute to either.

Comment: Done

Line 132:

Aetiologic and causative are mutually redundant. Would pick whichever author prefers.

Comment: Done

Line 133:

Thorn should be plural (thorns).

Comment: Done

*Acacia* should be italicized as a genus name.

Comment: Done

Line 134:

“The” before “mammal dung” is unnecessary.

Comment: Done

Line 136:

*Sordariales*, as a fungal order, should be italicized.

Comment: Done

Line 137-138:

“Still” is unnecessary in this sentence.

Comment: Done

Lines 140-143:

Suggest rewording for clarity. Ex: A recent study utilizing ITS2 metagenomics demonstrated the presence of multiple fungal species most commonly associated with soil and animal dung, including eumycetoma-associated agents, within BG samples obtained from patients living within the eumycetoma-endemic region of the White Nile.

Comment: Done

Lines 147-151:

Suggest rewording for clarity. Ex: To clarify the specifics of the epidemiology and environmental niche of the fungal “consortia” found within BG samples we undertook a study of soil and household samples from patients diagnosed and confirmed to have eumycetoma utilizing a similar metagenomic approach.

Comment: Done

Materials and Methods:

Lines 162-164:

Suggest adding reference to previous method utilized to identify eumycetoma pathogen (per Table 1 was metagenomics or culture but should be in body for clarity).

Line 169-173:

Suggest rewording for clarity and grammar. Important to maintain plurality with samples when listing and to italicize *Acacia*. Ex: Environmental samples were obtained from superficial soil (N=9) and soil at 30 cm depth (N=9), housing samples from walls (mostly made of soil and animal dung, N=7) and roofs (mostly made from local tree/plants, N=6), animal dung from domestic cows, goats, and donkeys (N=8), dried local plants generally fed to live stock (N=3), and thorns from *Acacia* species trees local to the area (N=7) (Table 1).

Comment: Done

Lines 175-177:

Suggest rewording for grammar and clarity. Ex: Additionally, soil samples were collected from non-endemic locations including Wagga village in Kassala State (N=3) and Port Sudan within Port Sudan State (N=2) (Table 1, Fig. 1).

Comment: Done

Lines 178-180:

Suggest rewording for grammar (many commas here but not many clauses or lists). Ex: The samples were collected utilizing sterile gloves placed into sterile labeled 50 ml Falcon tubes with a sterile spatula. Samples were then brought to the laboratory to be stored at 4 C before being portioned for both culture and DNA extraction.

Comment: Done

Line 183:

Comma after “media” is unnecessary. Can shorten “which included” to “including.”

Comment: Done

Line 188:

Dung and thorn can be singular.

Comment: Done

Line 192:

Oxford comma after “mycelia.”

Comment: Done

Line 196:

Suggest slight reword for grammar. Unclear of how “Still,” affects meaning and reading. Ex: Additional fungi unable to be identified by culture or morphology were identified by Sanger sequencing.

Comment: Done

DNAs can be replaced with “Nucleic Acids” for clarity and grammar.

Comment: Done

Line 199:

DNAs can be replaced with “nucleic acids” for clarity and grammar.

Comment: Done

Line 204:

As written should replace “was” with “were” as we are speaking of multiple species.

Could also be rewritten as “definitive speciation was determined by BLAST database query.”

Comment: Done

Line 207:

Suggest rewording “samples biodiversity” with “biodiversity within samples” to avoid confusing possessive tenses.

Comment: Done

Line 209:

Can remove comma after “cabinet.” Would reword end of sentence for clarity. Ex: “…and then resuspended utilizing sterile water…”

Comment: Done

Line 212-214:

This sentence is a bit confusing.

So, contamination was assayed by utilizing a blank control of DNA free water? That is, it was added as a separate sample then extracted and sequenced rather than added to a test sample (as this wording implies)?

Comment: Yes indeed

If this is the case, would suggest slight reword. Ex: Contamination between samples was excluded using Ultrapure DNA free water as a separate blank control.

Comment: Yes indeed

Line 222:

Can remove comma after “Library” and replace with “with.” Ex: Libraries were prepared using Nextera XT DNA Library with sequencing performed via the Illumina MiSeq Paired-End platform.

Comment: Done

Line 228:

Oxford comma after “merged”

Comment: Done

Line 230:

Suggest removing comma after parenthetical and adding “and”

Comment: Done

Line 232:

Spelling is “visualization”

Comment: Done

Line 239:

Can remove comma after “procedure”

Comment: Done

Results:

Lines 246-248:

Can remove comma after “samples” and replaced “and they” with “which”

Oxford comma between parenthetical and “and other species”

Comment: Done

Line 248:

Genus species shouldn’t be capitalized. Ex: *M.mycetomatis*

Comment: Done

Lines 250-251

Can change “and that” to “which”

Oxford comma between “*Bipolaris* (1.6%)” and “and *Chaetomium atrobrunneum*”

Comment: Done

Lines 253-256:

Suggest rewording for clarity and grammar. Ex: Several species of eumycetoma-associated fungi were found by ITS2 Sanger sequencing but not observed to grow in culture. These include…

Comment: Done

Line 260:

Should not capitalize metagenomics

Comment: Done

Line 263:

While controversial, all levels of taxonomy are starting to be italicized. Ex: subkingdom *Alveolata*. Should conform to NTD standards on the matter.

Comment: Done

Line 265-267:

Suggest splitting list into two separate sentences. Ex: As shown by the alpha rarefaction analysis (Fig. 2a), samples with the highest ASV diversity were from deep soil, housing walls, and superficial soil. This was followed by animal dung, housing roofs, thorns, and finally clinical BG samples.

ASVs and BGs here should be singular for grammar.

Comment: Done

Line 268:

ASV here should be singular.

Comment: Done

Lines 269-270:

ASV here should be singular.

Comment: Done

Line 273:

Spelling is visualized.

Comment: Done

There is a missing closed parenthetical for (based on the… Would suggest adding closed parenthetical after “ASV” as that seems to be the intent of the information included in the parenthetical.

Comment: Done

Lines 276-279:

Suggest rewording for clarity and grammar. Ex: Compared to total ASV count, ASVs of eumycetoma-associated fungi were found more commonly in housing roof (15%) and wall (13%) samples. This was followed by animal dung samples (11.6%), superficial soil and thorn samples (11.5% each), and deep soil samples (11%) (Tab. S1c).

Comment: Done

I believe that both superficial soil and thorn samples are meant to have 11.5% each rather than 11.5% collectively but is worth clarifying in text as baseline excel tables are very unfriendly to reader.

Comment: Done

Lines 281-285:

Suggest rewording for clarity and grammar, and to replace AVS with ASV.Ex: ASV metagenomics of samples obtained from the environment of endemic villages identified multiple genera associated with eumycetoma including….

Comment: Done

It is not clear per text if the environmental samples were from endemic villages, non-endemic locations, or both. Should clarify.

“in a lesser extent” should be replaced by “to a lesser extent”

Comment: Done

Commas should separate this list after “to a lesser extent”.

Comment: Done

It is not clear if both *Bipolaris* and *Acremonium* represented 0.01% of the ASVs associated with eumycetoma together or if both are 0.01%. Should clarify.

Comment: Done

Lines 287-289:

I am deeply confused by this sentence.

Does overall imply that it is across both environmental and clinical samples? And across both endemic and non-endemic environmental samples? Should clarify.

I believe what is meant is something like: *M.mycetomatis* represented 10% of all eumycetoma-associated sequences (0.7% of total sequences) across all samples within the study. This places it at the third most abundant eumycetoma-associated species following *Exserohilum rostratum* (28%) and *Alternaria alternatum* (14%).

Comment: Yes, indeed we rephrase the sentence according to the reviewer comment.

Lines 289-291:

Suggest rewording for grammar and clarity:

Ex: Interestingly of these three only *A.alternatum* were found to grow in culture (2%).

Comment: Done

Lines 292-296:

Another confusing set of sentences.

I believe what is meant is something like: On average patients affected by eumycetoma associated with *M.mycetomatis* had more *M.mycetomatis* found within their associated environmental samples when compared with patients with eumycetoma associated with other fungal genera (4% v 1%). However, this observed difference was not statistically significant (p>0.05).

Should clarify. And add specific calculated p value ideally.

Comment: Done

Fungi richness and diversity in soil

Line 299:

Suggest rewording for grammar. Ex: Richness of fungal diversity in soil

Comment: Done

Line 300:

Soil should be singular

Comment: Done

Lines 303-306:

Suggest rewording for grammar and clarity. Ex: 234 ASVs were found in superficial soil samples while 225 were found in deep soil samples with 27 (11.5%) and 25 (11.1%) being from eumycetoma-associated fungi respectively (Table S1b).

Comment: Done

Lines 307-312:

Suggest rewording for grammar and clarity. Are the hyphens representing a range or that one side is from superficial and another deep? If so, Ex: *M.mycetomatis* was the most common eumycetoma-associated species demonstrated in both superficial (0.79%) and deep (0.64%) soil samples. For superficial and deep soil samples this was followed by *Curvularia lunata* (0.61%, 0.5%), *E.rostratum* (0.34%, 0.3%), *Cladosporium* spp. (0.31%, 0.13%), and *A.alternatum* (0.3% each) respectively (Table S1c).

Example changed to add clarity to *C.lunata* naming, correct spelling of *Cladosporium*.

Comment: Done

Line 312:

Suggest adding “each” to parentheticals to clarify if observed percentages are for both deep and superficial. If they are a combination of both would add “together.”

Comment: Done

Line 313:

Would suggest write out full name of *Madurella fahalii* for clarity as is first time referenced in text.

Comment: Done

Lines 316-317:

Super interesting finding. Would consider adding a reason or speculation in the discussion about this. You would think metagenomics would have better resolution than Sanger and should be more sensitive than either Sanger or culture.

Comment: we added new consideration in discussion

Line 321:

Soil should be singular.

Comment: Done

Line 322: 348

Suggest separating sentences. Ex: …was detected in Kassala soil. In contrast to the endemic samples, ….

Comment: Done

Line 324:

Other should be singular.

Comment: Done

Line 326:

Spelling is *Cladosporium*

Comment: Done

Lines 329-331:

Suggest slight rewording for grammar and clarity. And utilizing PCoA to keep abbreviations standard from start of text. Ex: Greater beta diversity was noted on PCoA analysis amongst ASVs observed in soil samples from the endemic villages in White Nile and those in Port Sudan when compared with the non-endemic village of Wagga (Fig. 3).

Comment: Done

Fungal richness and diversity in walls and dung samples.

Line 333:

Suggest rewording for grammar and clarity. Ex: Richness of fungal diversity in wall and animal dung samples.

Comment: Done

Line 334:

“dungs” and “walls” should both be singular.

Comment: Done

Line 335:

“Dungs” should be singular.

Would suggest moving “of which” to after “23” to keep parallel with last clause in sentence. Ex: ….23 of which….29 of which…

Comment: Done

Line 338:

“dungs” should be singular.

Comment: Done

Line 341:

Spelling is *Cladosporium*

Comment: Done

Line 342:

Suggest changing “houses walls” to “housing wall” to avoid confusing possession v plural and assist read.

Comment: Done

Fungi richness and diversity in houses roofs and thorns samples

Line 348:

Suggest rewording for grammar and clarity. Ex: Richness of fungal diversity in housing roof and thorn samples.

Comment: Done

Lines 349-352:

Suggest rewording for clarity and grammar. Ex:

Nine roof and seven thorn samples were studied by metagenomics. 117 ASVs were noted in roof samples with 18 (15%) of them being associated with eumycetoma while 69 ASVs were found in thorns with eight (11.6%) of them being associated with eumycetoma (Table S1b).

Comment: Done

Suggest adding percentages to keep with general format of text. Rounding in keeping with NTD policy and author’s prior rounding conventions.

Comment Done

Lines 353-356:

Somewhat confusing set of sentences. I believe this is to show which genus-level and species-level IDs were found amongst fungal species ASVs in roof samples. Wording is not clear. If this is so:

“roofs” should be singular.

“species” should be changed to “genera” or “ASVs” to keep with idea of both sentences.

Would suggest changing “Among species” to “Amongst ASVs with species-level identification” for clarity.

Comment: Done

Lines 356-358:

Similar confusion here. If idea is to demonstrate genus and species level IDs:

Suggest separate sentences. Ex: In thorn samples the only eumycetoma-associated fungus to be identified to species level was *E.rostratum* (3%). However, multiple genus-level ASVs were found including…

Comment: Ok, we separated the 2 sentences

Lines 359-362:

Suggest rewording for grammar and clarity. Ex: Notably culture was only able to identify *A.niger*, *A.nidulans*, and *F.oxysporum* from thorn samples while *A.nidulans*, *A.niger*, *A.terreus*, and *C.atrobrunneum* were identified from roof samples. Interestingly, *C.atrobrunneum* was not observed in metagenomics analysis.

This is another super interesting finding with not finding *C.atrobrunneum* on metagenomics (presumably per text wording). Would attempt to expound on that in discussion if possible with *F.falciforme* observation above.

Comment: We added in discussion: Metagenomics provided a more comprehensive profile of the fungal species in the soil compared to culture-dependent methods and Sanger sequencing, demonstrating to be a powerful method for both eumycetoma diagnosing and identifying of fungi from environmental samples, even in the absence of growth. There was, however, an inconsistency, *C. atrobrunneum* and *F. falciforme* were only identified by culture and Sanger sequencing and were not detected by metagenomics. It can be attributable to the amplicon metagenomic method that may not be capable of amplifying all cultured fungi due to the biases associated with DNA extraction, PCR amplification and ITS primers. (DOI:10.1038/s41598-017-17132-4) This shows that the amplicon metagenomic approach based on the ITS2 region of fungal rDNA and the culture method are complementary.

Lines 363-365:

This segment seems to leave a bit unsaid regarding how frequently non-eumycetoma associated ASVs were observed statistically given percentages in the texts and ASVs. Would suggest either adding passages in your other samples expounding on this point or removing this passage. Favor the former but information may not be available. Could weaken your case or could provide evidence of new associations for other genera.

In light of the complexity of this study, we prefer not to include other information and to focus solely on eumycetoma-associated-species, the information about the 3 fungal species not associated with the disease is important to reinforce the hypothesis that F. senegalensis and Acremonium alternatum infections were taken out of the endemic villages. In fact, the 3 fungal species were found just in the sameBlack grains and not in the environmental samples

Fungi richness and diversity in black grains samples.

Line 367:

Suggest rewording for grammar and clarity. Ex: Richness of fungal diversity in black grain samples.

Comment: Done

Lines 368-373:

Suggest rewording for grammar and clarity. Ex: ITS2 amplicons previously obtained from BG samples from eumycetoma patients living in the endemic villages of Alsobahi (BG…., houses….) and El Andalous (BG…., houses….) were analyzed using the newest version of Qiime2 package….

Comment: Done

Lines 374-376:

Suggest rewording for grammar and clarity. Ex: The new analysis demonstrated similar results to prior analysis with an older Qiime2 package and found both eumycetoma-associated agents and non-associated agents….

Comment: Done

Lines 376-379:

Deeply confused about this passage. The second analysis found *Fusarium* not *Acremonium*? Are they not both associated with eumycetoma per the text? Worth expounding on in discussion as could be seen as a weakness to metagenomics approach. Likely inherent to two closely related fungi in terms of phylogenetics.

Suggest rewording for grammar and clarity. Ex: Interestingly, the identity of the fungi associated with one patient’s BG sample (house…) was found to be different on this reanalysis. On first pass the fungus was identified as *A.alternatum* while on second pass it was called *Fusarium* spp.

Would then address in discussion how both being in *Hypocreales* and being somewhat closely related might explain how this happened. Likely will undermine argument less than approaching it from a diversity of fungi in the sample perspective.

Comment: as commented up On first pass the fungus responsible of eumycetoma in patient 5 was identified as *Fusarium spp* [18], while on second pass it was called *Acremonium alternatum* (submitted on database in date 26-may-2021_[PRJNA732838](https://www.ncbi.nlm.nih.gov/bioproject/732838)), *Fusarium spp* and *Acremonium spp* belong to the Hypocreales order, so probable none of *Acremonium spp* sequences were present in the Metagenomics database during the first analysis.

These 2 eumycetoma associated species were detected in BG and not in the environmental samples of endemic region, as well as in not endemic, just few sequences of *Acremonium persicum* were detected,

While *Alternaria alternata* was detected in both endemic and not endemic villages

Lines 380-387:

Suggest rewording for grammar and clarity. Ex: All fungal species associated with eumycetoma were similarly detected on the new analysis with two notable exceptions. BG18 from a patient in house X failed to detect a previously characterized *F.senegalensis* while BG5 from a farmer in house Y failed to demonstrate a previously characterized *A.alternatum*. Further, three non-eumycetoma associated fungi were recovered in BG samples that were not found in environmental samples including *Sistotrema adnatum* (BG18), *Inocybe vulpinella*, and *Vishniacozyma* spp. (both BG5) (Table S1f, Fig. 4). Figure 4 visualizes the full range of fungi detected in BG samples.

Re-worded

Discussion:

Line 392:

Oxford comma after “communities”

Comment: Done

Line 396:

Suggest either replacing “that” with “it” or adding “gap” after “that” for grammar and read.

Comment: Done

Line 401:

Suggest separating “in general” with commas as a separate clause to parallel the second clause “,in particular,” Or removing commas from second clause.

Comment: Done

Line 405:

This may be an issue with the copy I received but is “metagenomics” in a slightly different color for everyone else? Is this an artifact of bolding? Very minute issue but is strange and noticeable on read.

Comment: sorry for that the color was grey that’s why its difficult to read

Lines 409-412:

Should be clear when speaking of environmental samples if we are talking about endemic or non-endemic context. This can affect your conclusion and could affect reader’s understanding.

Comma between “*Microascus”* and “*Bipolaris”* and Oxford comma after “*Bipolaris”*

Comment: Done

Lines 412-414:

Suggest rewording for grammar and clarity. Ex: Amongst environmental samples from endemic villages *M.mycetomatis* represented the first most common species recovered from all soil samples and third most common from dung and housing wall samples by metagenomic analysis.

Comment: Done

Lines 414-415:

Spelling is “findings”

Comment: Done

Would expound here on how that data supports it and speak about the thorn negatives. That is still a sticking point to me.

Would keep eumycetoma language for clarity. This study has nothing to do with actinomycetoma.

Comment: Done

Lines 417-420:

This is a very confusing passage. I believe you meant “”

Would reword, remove commas after “Sudan” and “river” and add “and” after “river.” Ex: The previous study demonstrated the occurrence of *M.mycetomatis* and other species associated with eumycetoma in the environment of the Southeastern villages of Sudan along the Nile River and confirmed their spatial distribution therein through regression and machine learning techniques.

Comment: Sorry you are right, we re-worded The present study showed the presence of *M. mycetomatis* and other eumycetoma-associated species in two While Nile endemic villages in Southeastern of Sudan, supporting the results of a previous study (2) that predicted the spatial distribution of eumycetoma in Sudan, including the While Nile region using regression and machine learning techniques.

Lines 423-425:

This is a very confusing passage. Multiple species of eumycetoma-associated fungi were found in environmental samples. *A.alternatum* was actually detected in multiple environmental samples from both endemic and non-endemic locales. It was not detected on the second round of metagenomics in one patient previously diagnosed with it through the first metagenomics round. Is that not accurate? While your implication is likely accurate for *F.senegalensis*, I do not believe the passage accurately represents your findings nor can it expand to *A.alternatum*.

Would keep eumycetoma language for clarity. This study has nothing to do with actinomycetoma. OK

Comment: We included the 3 fungal species non associated to eumycetoma, detected as F .senegalensis and Acremonium alternatum in BG 5 and 18 and not detected in the environmental in the discussion as follow:

The obtained data showed that fungal eumycetoma causative agents *Acremonium alternatum and Falciformispora* senegalensis and additional 3 species not associated to eumycetoma, detected in patient living in house 5 and house 18 respectivelly, were not detected in the environmental samples were not found in environmental samples. It can be extrapolated that eumycetoma infection in these patients was acquired outside the studied villages. That may be supported by the villagers' habit of continuous movement between different villages in the area due to socioeconomic reasons. However, further studies are needed to clarify this.

Line 427:

Would suggest removing “the” before “different” for grammar.

Comment: Done

Line 432:

ASVs not AVS

Comment: Done

Line 433-436:

Suggest rewording for grammar and clarity. Further interestingly *Fusarium* (0.4% of ASV sequences demonstrated in environmental samples from endemic/non-endemic villages) and *Aspergillus* species (X% of ASV sequences demonstrated in environmental samples of endemic/non-endemic villages) represented the most common species recovered utilizing culture techniques. The exact explanation for this finding is not clear.

Done including only endemic samples

Line 438:

Would then link this paragraph with the previous one as this passage seems to try to expound on why *M.mycetomatis* was not observed in culture. Ex: One possible explanation for this observation… OK

Lines 443-447:

Would reword for grammar and clarity. Ex: Another possible explanation for the difficulty of *M.mycetomatis* cultivation in vitro could be its ability to produce melanin and black grains. While this ability to escape immune responses may aid in its ability in producing such chronic infections in vivo, in vitro this may produce an effect that inhibits growth and sporulation similar to a bacterial biofilm or endospore. This idea could further be supported by the observation of hyaline molds and non-melanin containing fungi being more frequently isolated from both immunocompromised and malnourished patients than their dematiaceous counterparts.

Comment: accepted and rephrased according to the reviewer comment, thank you very much indeed

Lines 449-453:

Would reword for clarity and grammar. Ex: While the conclusions in this study may be limited by its small sample size it does offer some suggestions to reducing the disease burden felt by those living in endemic and often impoverished areas. These include using non-endemic soil and avoiding animal dung in housing construction and the use of proper footwear and gloves in agricultural and other activities that may facilitate traumatic inoculation.

Comment: Done

Lines 454-457:

Confusing wording here. Would stick with metagenomics even if NGS was involved for clarity.

Suggest rewording last two sentences together for clarity and grammar. Ex: While metagenomics proved very useful in assaying environmental and clinical samples towards the goal of greater understanding of the connection between environmental fungi and eumycetoma pathogens implementation of such techniques are still currently limited by prohibitive financial cost.

Comment: Done

Reviewer 3:

Reviewer #3: The article “Metagenomics detection of eumycetoma causative agents within eumycetoma patient’s household environment in two Sudanese endemic villages, in White Nile State” by Antonella Santona et al. aims to provide the epidemiological information about the source of causative agents for mycetoma. Using the metagenomics approach, the previously unidentified causative species might be easier identified when compared to the detection by cultured dependent methods. Assuming that this is the study’s novelty, however, this point was not clearly stated in the introduction part of the article. For the method, the study was designed based on a previously published study site, so the sample size might be too small to answer the question at large and might not be able to apply in the other endemic area, which leads to the lack of the external validity of the study. However, the information might be used in endemic areas with similar environmental factors. The authors should discuss this limitation more.

Comment: we add this part as a limitation in our discussion. Furthermore, this study was the first ever.

The method of metagenomics analyses is well described and is enough to answer the research questions. However, several analyses are possible to be added by using the existing data set to create a more sophisticated article, for example, the rarefaction analyses of each sample, the comparison of the phylogenetic diversity (PD) between each sample, especially from endemic vs. non-endemic areas, or other kinds of alpha diversity such as Chao1, etc. Please consider adding these analyses.

Comment: Done

One additional weak point of this study is the lack of data about the abundance of the mycetoma-associated taxon in other type of samples in the non-endemics area except soil (e.g., roof, water). So, it is hard to conclude that the present of the mycetoma-associated taxon in each type of sample in endemics areas might are the indeed source of the infection. The authors should consider adding this part of experiment or discuss about this limitation.

Comment: We specify better in the text as explained above in previous answers

The other concern of the method is the availability of the sequencing data, which the authors failed to demonstrate or still do not deposit on any repository (for example, GenBank).

Comment: Sequences were uploaded on genebank

Reviewer #3: Most of the result are clearly presented according to analysis plan and the pipeline of metagenomics analysis. However the results is not clearly presented and several important imformation is still lacking, for example most of the results are presented in the abundance of the mycetoma-associated taxon in each sample pattern. However, the abundance of the mycetoma-associated taxon in the endemic area compared to non-endemic regions should be included and shown by using a statistical calculation to prove that the increase in the percentage of the mycetoma-associated taxon is genuinely associated with the endemicity of the disease. Most of the study is still lacking in these essential results (as mentioned in methods), or some sample was already done (soil) (for example, lines 318 – 320), but the statistical analysis has still lacked. Therefore, an additional analysis of this dataset is needed.

In addition to the analysis, the table and figures also should be revised. In table 1, the name of sample types should be revised (what are the meaning of S1, S2, W,…?). In figure legends, please describe the meaning of abbreviations such as K soil and PS soil. Please considers adding color to the beta-diversity PCoA plot (Figure 3) and add a sub-figure that extracts only the information about soil, especially to demonstrate the difference between endemic and non-endemics areas.

Comment: Done

Reviewer #3: The public health relevance was clearly addressed in the discussion and conclusion part. But the limitation of the study should be more focused than the small sample size. The discussion has required revision; for example, what is the importance of the presence of each mycetoma-associated taxon or the possible route of infection from the source by citing the previously published data or related to the local activity which not clearly illustrated for a general audience. Lastly, the conclusion and suggestion of the study, such as changing house building material, might be a slightly over conclusion because many infection risk factors are required to identify. So the conclusion might be that “the soil in the endemic area might be the source of infection” would be more suitable.

Comment: Done and added

Reviewer #3: Overall, even though the study still has a big room for improvement, but the information from this study might be an important key for the prevention and control of the disease. The reanalyses of the data is needed. Moreover, there are a major concern about the ethical issues in this study for example the use of isolated specimen from human without state about the IRB approval, and the availability of sequencing data.

Comment: The ethical part was added in the methodology, for the availability of the Sequences were uploaded on GenBank with the BioProject ID: PRJNA851016. We added in the text
